# Dietary interventions with or without omega-3 supplementation for the management of rheumatoid arthritis: a systematic review protocol

**DOI:** 10.12688/hrbopenres.13136.1

**Published:** 2020-10-02

**Authors:** Tala Raad, Elena George, Anne Griffin, Louise Larkin, Alexander Fraser, Norelee Kennedy, Audrey Tierney

**Affiliations:** 1 Discipline of Dietetics, School of Allied Health, Faculty of Education and Health Sciences and Health Implementation Science and Technology Centre, Health Research Institute, University of Limerick, Limerick, Ireland; 2 Deakin University, Institute for Physical Activity and Nutrition (IPAN), School of Exercise and Nutrition Sciences, Geelong, Victoria, 3220, Australia; 3 Discipline of Physiotherapy, School of Allied Health, Faculty of Education and Health Sciences, Implementation Science and Technology Centre, Health Research Institute, University of Limerick, Limerick, Ireland; 4 Department of Rheumatology, University Hospital Limerick, Limerick, Ireland; 5 Graduate Entry Medical School, Faculty of Education and Health Sciences, University of Limerick, Limerick, Ireland

**Keywords:** Rheumatoid arthritis, diet, omega-3, randomised controlled trials, non-randomised controlled trial, systematic review, protocol

## Abstract

**Background: **Rheumatoid arthritis (RA) is an autoimmune disease characterised by swollen and painful joints. It is hypothesised that changes in lifestyle factors such as consuming a healthier diet may  reduce the severity of RA symptoms. People living with RA commonly make alterations to their dietary intake with the hope of improving their symptoms. This systematic review aims to discuss the effects of dietary interventions with and without omega-3 supplementation for the management of rheumatoid arthritis.

**Methods:** A systematic review of randomised controlled trials (RCTs) and non-randomised controlled trials (NRCTs) will be conducted. MEDLINE, EMBASE, The Cochrane Library (Cochrane Database of Systematic Reviews, Cochrane Central Register of Controlled Trials (CENTRAL), Cochrane Methodology Register) and CINAHL will be searched from inception without using date restrictions. Primary outcomes will include measures of disease activity, inflammation and quality of life among adults living with RA. Study selection will follow the Preferred Reporting Items for Systematic Reviews and Meta-Analyses (PRISMA) guidelines, and the methodological appraisal of the studies will be assessed independently by two different reviewers (TR and AG) using the Cochrane Risk-of-Bias Tool for RCTs, and Risk-of-Bias In Non-Randomised Studies Tool for NRCTs.

**Ethics and dissemination: **Ethical approval is not required for this systematic review. Only publically available data from previously published studies will be used. The findings of this systematic review will be submitted for publication in a peer-reviewed journal and presented at relevant conferences.

**PROSPERO registration:** CRD42020147415 (11/02/2020).

## Introduction

### Background

Rheumatoid arthritis (RA) is a chronic autoimmune inflammatory disease that primarily affects the joints of the hands and feet, causing malformation and damage to these joints ^1^. It is one of the most prevalent forms of inflammatory polyarthritis, affecting almost 1% of the adult population worldwide ^2^. RA is three times more common in women than in men and although the typical age of onset is between 40–50 years, it can occur at any age ^3^. The quality of life of people with RA is generally severely impacted due to the increased risk of morbidity and mortality associated with the disease ^4^. People with RA normally undergo lifelong pharmacological treatment to delay the progression of the disease and to ease symptoms, which include pain, fatigue and morning stiffness ^5^. Despite the increasing number of new treatments, complete remission of the disease has not been achieved for people living with RA and therefore treatment strategies that work alongside medication are required for the management of the disease ^6^. Non-pharmacological treatments include physiotherapy as well as lifestyle and behaviour change counselling to achieve smoking cessation, optimising diet and exercise ^7, 8^.

Nutrients and dietary patterns have major influences on human health ^9, 10^. However, the evidence on the association between diet and rheumatic diseases is weak and inconsistent ^11^. People living with RA often report an improvement in their symptoms following certain types of dietary patterns or by removing certain foods from their diet ^12^. It is thought that these improvements may be achieved via a decrease in inflammation, improvement in lipid profiles, enhanced intestinal flora and/ or an increase in antioxidant levels ^13, 14^. People living with RA are often eager to experiment with various dietary approaches ^15^ to alleviate symptoms and improve quality of life. Studies with interventional and prospective observational designs have provided valuable insight and evidence on the topic. The most commonly reported diets that have been trialled in RA include vegetarian, vegan, Mediterranean, elemental or elimination diets ^16^. A recent review provided evidence on the benefits of a Mediterranean diet for increasing physical function and reducing pain in people with RA ^17^. Adherence to gluten-free and elemental diets have also been directly linked to improvement in disease activity ^18^ and a vegetarian dietary pattern was associated with clinically relevant long term improvements in symptoms of the disease ^19^.

Despite a small number of studies assessing the effect of diet, the majority of the evidence in this area focuses on the role of dietary supplementation rather than specific dietary patterns. Predominantly, studies have evaluated the role of omega-3 fatty acids in the treatment of RA ^20^. Omega-3 fatty acids, a component of fish oils, are reported in a number of studies to have beneficial effects relating to human health and disease ^21^. Many intervention studies found associations between intake of omega-3 fatty acids and various conditions including lower rate of cardiovascular diseases, inflammatory bowel disease, asthma, cystic fibrosis ^22^. In RA, there is a great body of evidence for the therapeutic benefits of omega-3 fatty acids, improvements are thought to be related to a mechanism involving the modulation of the immune system which decreases the action of inflammatory compounds ^23^. The majority of the studies have shown that daily supplementation with omega-3 fatty acids could result in significant clinical benefits and improvement in many outcome measures in RA ^24^. While there is some evidence that dietary patterns may be beneficial for managing the symptoms of RA, the results continue to be contradicting and inconclusive and are yet to identify a particular dietary pattern that is helpful in the management of RA. Furthermore, the effect of dietary interventions with or without omega-3 supplementation for the management of this chronic disease has not been established and thus, a review of cumulating evidence in this area is warranted for better understanding.

### Aim and objectives

The aim of this systematic literature review is to explore the evidence for the effectiveness of dietary interventions with or without omega-3 supplementation in improving disease activity, inflammatory status, physical function and quality of life in adults living with RA. Specific objectives include: (1) collate the current evidence base in the area of diet and RA; (2) explore the effects of dietary interventions with or without omega-3 supplementation in the management of RA; (3) explore associations between dietary interventions and health outcomes in people with RA, specifically (a) inflammation, (b) disease activity, (c) physical function and (d) quality of life.

## Methodology

This protocol has been prepared following the Preferred Reporting Items for Systematic review and Meta-Analysis Protocols (PRISMA-P) guidelines ^25^ as shown in the PRISMA-P checklist (see *Reporting guidelines* ^26^). The protocol has been registered with the international prospective register of systematic reviews (PROSPERO) database for registration [ CRD42020147415, 11/02/2020].

### Eligibility criteria

***Types of studies*.** For the purpose of this systematic review, we propose to include all controlled trials (randomised and non-randomised) reporting outcome measures at pre- and post-intervention. Only peer-reviewed publications in the English language will be eligible for inclusion and no restrictions on publication year will be applied. Studies with other methodological designs (e.g., cohort, case-control, cross-sectional, etc.), and study protocols and conference abstracts will be excluded.

***Population*.** The systematic review will include studies with participants aged 18 years and older who have a definite diagnosis of RA as per the American College of Rheumatology (ACR) criteria ^27^.

***Types of interventions*.** Studies conducted among adults with RA who received any type of dietary intervention alone or those that included a dietary intervention with omega-3 supplementation will be included. Studies on other rheumatic diseases (e.g. lupus, osteoarthritis) will be excluded but studies of mixed populations that include RA will be included where RA data is reported separately. Studies on dietary supplementation only and studies assessing the risk of development of the disease are beyond the scope of this systematic review. Furthermore, studies where the dietary intervention was part of a multimodal intervention (e.g. with exercise) or includes other co-interventions will not be included. Interventions of any duration will be included (e.g. short-term or long-term).

***Comparators*.** We will include studies comparing the effects of a dietary intervention with usual or a control diet. Studies comparing more than one dietary intervention will also be included.

***Types of outcome measures*.** We are interested in both clinician and patient reported outcome measures, assessed by either subjective or objective indices (e.g. blood, urine, questionnaire, food diaries). The primary outcomes of interest are the effects of dietary interventions with/without omega-3 supplementation and disease activity, inflammation, physical function and quality of life. The secondary outcome measures will include reported pain, fatigue and participants’ overall experiences of the effects or impact of diet alone or with Omega-3 supplementation.

### Search strategy

A systematic literature search will be conducted using the following databases: MEDLINE, EMBASE, The Cochrane Library (Cochrane Database of Systematic Reviews, Cochrane Central Register of Controlled Trials (CENTRAL), Cochrane Methodology Register) and CINAHL. The title and abstracts retrieved from the electronic databases and references will be exported to EndNote bibliographic software for storage and the removal of duplicates. After removal of duplicates, the title and abstracts will be exported to Rayyan software for reviewing. Two independent reviewers will evaluate the articles returned by the searches against the inclusion and exclusion criteria. Any disagreement over eligibility will be resolved through discussion with a third reviewer. The reference lists of identified publications will be searched to identify additional relevant publications.

All initial searches will be performed by TR and publications will be organised in reference manager Endnote. The search strategy will be developed in consultation with an academic librarian at the University of Limerick. No limits will be applied on publication year. Articles will be first excluded on the basis of title and abstract against inclusion and exclusion criteria. Full text of studies that appear eligible on the basis of their abstracts will be obtained, read and evaluated by two independent reviewers. Conference reports and abstracts will be excluded. The literature search strategy is included as *Extended data* ^26^.

### Data extraction

Two reviewers will extract data from included studies independently. The information to be extracted includes the following: study design, study geographic location and setting, participants’ age and gender, sample size, disease duration, type and duration of intervention, type of comparators, outcomes measured, diet compliance, dosage of Omega-3 supplementation, follow-up and results of the intervention. In the event that clarification or additional information is required, every attempt will be made to contact the authors to collect the missing data.

### Quality assessment

The Cochrane risk of bias tool from the Cochrane Handbook for Systematic Reviews of Interventions V.5.1.0 will be used to assess the quality of the included studies ^28^. The following aspects will be evaluated: (1) randomisation sequence allocation; (2) allocation concealment; (3) selective reporting of outcomes; (4) blinding of participants and personnel; (5) blinding of outcome assessment; and (6) incompleteness of outcome data. The risk of bias for each outcome across individual studies will be classified into low, high or unclear risk of bias and summarized in a table. Two reviewers will independently evaluate the quality of included articles. A third reviewer will resolve differences or if consensus needs to be achieved.

### Data synthesis

We anticipate that the nature of this review will be largely descriptive; we are interested in the effects diet with or without omega-3 supplementation may have on key outcomes measured among adults with RA. Data from published studies will be collated and summarised. Difference in outcome measures between groups and change between groups will be reported, depending on the analysis reported for individual studies We anticipate a limited ability to conduct a meta-analysis for this review due to the inherent variability across dietary interventions, differences in the measurement of outcomes and how populations are selected. However if a meta-analysis can be conducted the authors will use a random effects model to assess the effect of a diet or supplementation on an outcome measure in RA.

### Potential limitations

The proposed systematic review may include non-randomised trials that may have a higher risk of bias and may yield evidence of low certainty. Language restriction to English only is another possible limitation for the proposed paper. Additionally, there may be study bias due to the nature of dietary intervention studies and inability to blind study participants.

### Ethics and dissemination

This systematic review involves secondary analysis of existing data and therefore, does not require ethical approval. The findings of this research will be submitted for peer-reviewed publication in an open access academic journal, and presented at relevant conferences.

### Patient and public involvement

Patients and public will not be involved for the purpose of this study. However, we expect that the findings of this systematic review will assist in the execution of a pilot dietary intervention with a patient and public involvement (PPI) component.

### Amendments to protocol

We will carefully report any changes and update PROSPERO should the protocol be amended.

### Study status

The study is currently ongoing. The expected end date for the study is December 2020.

## Conclusion

The proposed systematic review will synthesize existing evidence to determine the effects of dietary interventions with or without omega-3 supplementation for the management of RA in an adult population with aims to strengthen and update the current evidence base. Results from this systematic review may inform and facilitate the development of dietary guidelines and recommendations for people living with RA.

## Data availability

### Underlying data

No underlying data are associated with this article.

### Extended data

Open Science Framework: Dietary interventions with or without omega-3 supplementation for the management of Rheumatoid Arthritis: A systematic review protocol. https://doi.org/10.17605/OSF.IO/6J8ZR ^26^.

This project contains the following extended data:

- Supplementary file 1 (search strategy)

### Reporting guidelines

Open Science Framework (OSF): PRISMA-P checklist for “Dietary interventions with or without omega-3 supplementation for the management of rheumatoid arthritis: a systematic review protocol”. https://doi.org/10.17605/OSF.IO/6J8ZR ^26^.

Data are available under the terms of the Creative Commons Zero “No rights reserved” data waiver (CC0 1.0 Public domain dedication).
