# Supplementary material for: Dietary interventions with or without omega-3 supplementation for the management of rheumatoid arthritis: a systematic review protocol
Source: HRB Open Res. 2020 Oct 2;3:72. [Version 1] doi: 10.12688/hrbopenres.13136.1 (PMC8138488; doi:10.12688/hrbopenres.13136.1)
Supplement: Peer review history

# Peer review: Reviewer response for version 1

**DOI:** 10.21956/hrbopenres.14248.r29267

**Published:** 2021-05-20

**Authors:** Naheed Aryaeian

**Affiliations:** 1 Department of Nutrition, School of Public Health, Iran University of Medical Sciences, Tehran, Iran

Dear editor,

 This topic is a good and application subject, it has been written in a good manner, but I have some comments as following:

 This systematic review aims to discuss the effects of dietary interventions with or without omega-3 supplementation, but there are many dietary interventions (such as fasting, vegetarian, meditation, elemental…) in this diseases that controls them as confounding factors is hard. It was better the topic and aim of the study were “The effect of omega-3 supplementation for the management of rheumatoid arthritis: a systematic review protocol"

 Data analysis needs more explanation.

 It was better to mention mesh keywords.

 An Introduction has been mentioned "While there is some evidence that dietary patterns may be beneficial for managing the symptoms of RA, the results continue to be contradicting and inconclusive and are yet to identify a particular dietary pattern that is helpful in the management of RA” but the study included w3 supplementation, not dietary patterns.

 Regards,

Is the study design appropriate for the research question?

Yes

Is the rationale for, and objectives of, the study clearly described?

Yes

Are sufficient details of the methods provided to allow replication by others?

Yes

Are the datasets clearly presented in a useable and accessible format?

Yes

Reviewer Expertise:

Nutrition.

I confirm that I have read this submission and believe that I have an appropriate level of expertise to confirm that it is of an acceptable scientific standard.

# Peer review: Reviewer response for version 1

**DOI:** 10.21956/hrbopenres.14248.r29125

**Published:** 2021-04-12

**Authors:** Janet L Funk

**Affiliations:** 1 Department of Medicine & Department of Nutritional Sciences, University of Arizona, Tucson, AZ, USA

The authors will perform a systematic review of the literature to identify and summarize randomized clinical trials testing effects of dietary interventions alone or in combination with omega-3 fatty acid supplementation in ameliorating rheumatoid arthritis endpoints related to inflammation, disease activity, physical function and quality of life. Five data bases will be searched, including Cochrane Central, Articles will be included but conference reports and abstracts will be excluded. While the reviewer was not able to access the complete search strategy deposited in OSF, the brief strategy outlined in PROSPERO appeared appropriate, although the inclusion of alternate and/or more specific terms terms, which may be planned, could aid in complete discovery (e.g., marine oils, fish oils, Mediterranean diet). Methods of assessing citations for inclusion are appropriate.

 Major Concerns:

1. Some relevant recent reviews in this area were not cited (e.g. systematic review of marine oil supplements in RA ^1^ **, **review of Mediterranean diet and fish oil in RA ^2^), raising some concerns about the scientific context in which the study was planned and will be presented once completed. Nonetheless, results will be novel. 
2. While a systematic review of marine oils in RA was published in 2017, interpretation of clinical trial data to be summarized here, examining effects of diet alone or diet in combination with omega-3s may be difficult to assess and somewhat incomplete when trials examining omega-3s alone with "standard" diets are excluded. This will be a limitation.

Minor concerns:

1. A strategy for including or excluding an RCTs found via Cochrane databases for which data have been made public via clinical trials.gov but not otherwise published or analyzed needs to be established.
2. In addition to extracting data on omega-3 doses, as planned, consideration of specific omega-3 supplement content should be included (i.e. EPA vs DHA, and possible inclusion of other bioactive but non-omega-3 content in supplements), although in some cases it is also possible that this may not be described.
3. Some dietary approaches are intended to increase omega-3 intake. If available, total omega-3 dosing via the diet should be extracted as well.
4. Race was not listed as part of data extraction but should be included as it may influence responses to omega-3 supplementation or dietary approaches intended to increase omega-3 consumption (e.g. due to FADs genotypes).

Is the study design appropriate for the research question?

Yes

Is the rationale for, and objectives of, the study clearly described?

Yes

Are sufficient details of the methods provided to allow replication by others?

Yes

Are the datasets clearly presented in a useable and accessible format?

Yes

Reviewer Expertise:

In vivo metabolism, active metabolites and bone protective bioactivity of dietary polyphenols consumed as supplements, including those present in complex plant-derived extracts.

I confirm that I have read this submission and believe that I have an appropriate level of expertise to confirm that it is of an acceptable scientific standard.

## References

[1] : Marine Oil Supplements for Arthritis Pain: A Systematic Review and Meta-Analysis of Randomized Trials. *Nutrients*.2017;9(1) : 10.3390/nu9010042 10.3390/nu9010042 28067815PMC5295086

[2] : Role of Diet in Influencing Rheumatoid Arthritis Disease Activity. *Open Rheumatol J*.2018;12: 10.2174/1874312901812010019 19-28 10.2174/1874312901812010019 29515679PMC5827298
